# “The City of the Hospital”: On Teaching Medical Students to Write

**DOI:** 10.1007/s10912-015-9348-2

**Published:** 2015-07-17

**Authors:** David J. Hellerstein

**Affiliations:** Department of Psychiatry, New York State Psychiatric Institute, Columbia University, 1051 Riverside Drive, Unit #51, New York, NY 10032 USA

**Keywords:** Medical student education, Creative nonfiction, Writing workshop, Personal narrative, Medical humanities

## Abstract

“The City of the Hospital” is a creative nonfiction writing workshop for medical students, which the author has conducted annually since 2002. Part of the required preclinical Narrative Medicine curriculum at the Columbia University College of Physicians and Surgeons, this six-week intensive workshop includes close readings of literary works and in-class assignments that are then edited by fellow class members and rewritten for final submission. Over the years, students have produced a wide range of compelling essays and stories, and they describe the class as having an effect that lasts throughout their further medical training. This special section includes selected works from class members.

## Introduction

For well over a decade I have been teaching medical students to write. At first it was an odd assignment for me—a research psychiatrist at the New York State Psychiatric Institute who conducts clinical trials—to face a room full of skeptical second year medical students, few if any of whom aspired to a literary career.

As part of their training, medical students at Columbia University College of Physicians and Surgeons are required to take a six-week class in ‘Narrative Medicine’ (Charon 2006). They choose among options as varied as “The Philosophy of Death,” “Social Justice and Health,” “Gender and Illness Narratives,” fiction and poetry workshops, “Narrative Photography,” “Mindfulness Meditation”–and my creative nonfiction writing class, “The City of the Hospital: The Medical Student as Writer.” But they can’t opt out: Narrative Medicine is a required course.

The goal is to train better doctors by exposing medical students to humanities perspectives at a crucial phase of their education–before they actually start working on the wards. *How is that possible?* one might reasonably ask. *If they're not well-rounded, ethical people by the time they are admitted to medical school, how can a brief course possibly make a difference?* Plus, why bother? Given the explosive growth of medical knowledge, shouldn’t they be spending all their time learning physiology, pharmacology, molecular biology, and biochemistry, things that will be essential to their future as physicians?

And yet, the very inclusion of Narrative Medicine classes in the packed Columbia Physicians and Surgeons curriculum is a powerful statement in itself: that the humanities have value in a daunting world of fact-based knowledge and that they can provide something essential to the life of a doctor.

Truthfully, it was not entirely a surprise that I was selected to teach this class, since for several decades of being a physician, administrator and researcher, I have juggled a parallel career as a literary writer, publishing several books—including fiction, nonfiction and memoirs—as well as writing for numerous literary and journalistic publications. But until then I had always been an outsider; my writing activities were pursued during off hours, stolen time. This was the first time I had been invited to be part of the medical curriculum.

For “The City of the Hospital,” I was assigned a dozen students, and we were scheduled to meet for three hours each Tuesday afternoon for a crash course in writing creative nonfiction. I asked Dr. Charon to assign me students who wanted to be there; it seemed too difficult to try to rope in the unwilling. I couldn’t bear the idea of teaching a dumbed-down curriculum (“Tell your patient’s story in his or her words”). And thank God I wasn’t being asked to give one of the all-too-sadly needed classes in ‘writing in the electronic health record’ (i.e., how to type something reasonably coherent in the lonely free-text fields set among thickets of check-boxes and numerical data-fields)—both of these necessary because too many medical students never learn to write basic American prose. Instead, I opted from the beginning to teach a true writing class, challenging each student to incarnate herself as a serious literary writer for those six weeks, to put the best of that self on each page.

Probably the best moment each year is when my puzzled students read Isaac Babel’s short story, “My First Goose” (2006) and excerpts from Italo Calvino’s *Invisible Cities* (1978). They stare at me, deer in the headlights.

*Why are you making us read these things? What do these have to do with becoming a doctor?*

Hemingway’s story “Indian Camp” (1995), yes, they can understand what that has to do with medicine, as it recounts a young boy’s journey with his surgeon father to deliver a baby at a remote logging camp; similarly William Carlos Williams’ “The Girl with a Pimply Face” (1984), a presumably autobiographical story of an inner-city physician’s efforts to help an immigrant family. But Babel writes of a bookish Jewish college graduate dropped into a company of illiterate Cossack cavalrymen in the chaos of the Russian Revolution, and Calvino transports the reader to medieval Venice to listen to the explorer Marco Polo’s fanciful dialogues with the elderly Emperor Kublai Khan, spinning tales of impossible cities.

*What’s your point?* they wonder. And, more worriedly, *What do you expect from us?*

It is a literary boot camp, a crash course in being a writer, six long afternoons during which we review readings, then do in-class writing exercises for forty-five minutes or so, and then pick several ‘volunteers’ to read aloud. Squirm as they might, they must read their raw work aloud to their classmates.

“Anything you write here is fair game,” I tell them, “You can’t say no.”

Over the years, class members have included everything from accomplished poets and short story writers to total novices, biophysics majors and humanities-innocent engineers. In recent years, I’ve noticed an increasing diversity of students—gender, ethnicity, nationality, and culture, including students born in Vietnam, Nigeria, Colombia, and East LA, not just the usual suburbs of New York, Boston, and Philadelphia—and a comparable broadening of perspective and life experience.

Some of them gradually admit having the ambition to follow in the pathway of Atul Gawande or Oliver Sacks, a yearning to write elegantly and compellingly for literary audiences. For those aspiring physician-writers, the challenge they face is formidable: their training experiences are shared by tens of thousands of other young people each year. The annual number of graduating American medical students is about 17,000, and there are 850,000 physicians practicing in the US and perhaps 8 to 10 million doctors in the world. They are hardly unique.

“Everyone in this room,” I tell them, rubbing it in, “will cut open a cadaver, everyone will do a first pelvic exam and participate in the delivery of a baby and watch someone die–watch *many* people die.” They will be among crowds of thousand who observe amazing medical rescues and terrible, avoidable mistakes. At the same time, I tell them, they are explorers in the ever-new city of the hospital. Like anthropologists, they are both observers and participants, modifying their environment with their very presence. The city of the hospital—this new, ever-evolving city which has had millions upon millions of previous inhabitants but of which they are the first new explorer—is an amazing world in which they are privileged to spend the rest of their lives (Hellerstein 2004).

“How can you make it new? What can you say that is different from the thousands who have been here before and who will be here in the future? What is it that you observe that is truly unique and that at the same time illuminates general truths?”

It is a daunting challenge, but really, no different than what any writer has always faced.

And so, each year there’s the shock of silence when we sit in the room with laptops and iPads fired up–and no one moves. I watch them thinking, whispering, even texting: *He can't be serious, wanting us to write for so long!* They are used to five-minute free-writes following an instructor’s prompt. But forty-five minutes seems an eternity. After interminable sighing, staring, quiet moaning, the keys start clicking, pens start scratching, so by the end of the period, when I call out, “Put your pens down, close your computers!,” there’s an audible protest against the return to the mundanity of the classroom.

*

But *why* write? I need to demonstrate to them, a rightly skeptical audience, that the narrative method is a valid method in its own right. Narrative is so different from other methodologies they study in class—statistical techniques, laboratory methods, imaging technologies, epidemiological sampling—the innumerable disciplined ways of viewing the world in which they will be immersed over the coming decades. It is different too from the journalistic method; it is not just reporting but a way of writing that explores beyond the limits of objectivity. Why *narrative*? As venerable as it may be, I contend to them, narrative is yet capable of revealing new truths about medicine that can be communicated to the outside world.

In brief, the world of medicine they face—and will be facing over the half-century of their careers—is a *new* New World. What they can observe in their day-to-day work truly is ‘news,’ much more than what is Tweeted or Facebooked or flashed in newspaper headlines or on the TV news. I expect a lot from them, regardless of their prior experience. There are always one or two jokers, making sarcastic asides, browsing their email, but over the weeks the razzers often become the most seriously engaged.

As you will see from the essays and stories that accompany this essay, the writing that emerges from this process is often incredibly intense and moving, surprisingly expertly done, given that it is composed in forty-five minute bursts. Often the best work comes from those with the least prior experience–the biophysics majors, the dedicated molecular biologists and radiologists or orthopedists-to-be. Their output is often crude at first but rises on a steep learning curve over the six-week course: these are really smart kids (and so young, to boot!); I guess that says something about the rigor of the medical school admission process. I ask them to write about “an initiation,” and they end up with pieces about flying off ski jumps, piloting a small plane, being branded with a red hot broom handle during a fraternity hazing. Such pieces have nothing to do with medicine, but I'm happy with those since they are fresh, authentic, pulsing with life.

The following week I ask them to write about their experiences with medical technology. Then, they write about their years of premedical life, so often extended these days, and they tell moving stories about working in the cardiology lab with dogs who are going to be sacrificed or with genetically modified mice with fluorescent neurons who likewise will die for science. They write about working as hospital volunteers in the Bronx, as paramedics in the New Mexican desert, as observers in remote clinics in rural India where the electricity goes out in the operating room in the midst of cataract surgery. Or in a remote leper colony whose inhabitants ‘crawled to the doctor’s hut seeking rice wine to help soothe their sore appendages.’

And then, invoking the spirits of Italo Calvino and Jorge Luis Borges, I ask them to write about their early experiences in the city of the hospital. They write about the ID badges that we medical personnel use to get past the various barriers/doors/entrances of the hospital complex in order to enter the medical library, dorms, ICUs, surgical suites. They write about the mysterious way in which people always are arriving at the hospital on foot, in wheelchairs and stretchers, by taxi and ambulance and private car, but how the departure of those whose lives end here is unseen, how the dead disappear so invisibly that they could be entirely vaporized.

Inspired, they write of following a rolling gurney through creepy underground tunnels on its way to the radiology suite where their anatomy class cadaver is to be CT scanned to show the disease that their scalpels will later reveal. They write a fanciful David Foster Wallace-esque ‘laboratory manual’ for doing EEGs with screaming young children. They calculate the odds that the man in front of them could have had the particular complications that have led him to be in hospital: less than 1 in 3 billion; yet all the patients on the floor have 100 % chance of having an illness even if individually they were a thousand times more likely to have been struck by lightning. We see portraits of irritable neurosurgeons cursing when instruments are missing, of bagel-bringing anesthesiologists whom all residents look forward to assisting, and of innumerable kindly patients letting med students poke them, listen to bowel sounds, try to evoke reflexes.

Astutely, they pick up on the surrealism of the hospital city: crafting a nonfiction piece inspired by graphic novels, with five quick acts of an experience with illness, in which the City of the Hospital is personified as a complex, even diabolical character. And a phantasmagorical tale of a medical student riding on the A train, imagining the hospital as subway car, or perhaps the subway car as a hospital–each passenger experiencing an illness, even dying before his eyes, envisioning the mortality of fellow riders so differently from their classmates who are bankers or lawyers or teachers.

Most movingly, my students write about their own experiences with illness and loss during medical school, the cruel illnesses of grandmothers and childhood friends, their own harrowing brushes with Crohn’s Disease and cancer. In all, they write of their first realizations that they are hatching new selves as doctors, as healers, new identities in which they must come to terms with no longer being civilians.

From the pieces they write in the first five weeks, I ask them to pick one; it is given to another student to edit, then returned to the author to revise, and then to submit to me as a finished essay. Every year, several write publishable and striking work. To mention two: Sarah Chambers’s piece “The Harvest Team,” (2004) a moving tale about transporting a heart removed from a dying girl to be sewn into the chest of a young boy. And “Saturday Night in Mariposa,” by Brendan O’Byrne (2007), about his experiences as a paramedic in a national park, trying to resuscitate a man whose van rolled off a mountain road.

*

Like all teachers I prefer to think that I’ve had an impact, even on those who don’t fancy themselves to be writers. Could our hours here perhaps change how they how they converse with patients? Could it make them aware at 3 AM, attending a patient in the ICU, of the extraordinary nature of their experiences? Could it make them more sensitive to how their patients or families may be experiencing illness, so they might yearn not just to be excellent technical physicians but also humane ones?

It seems difficult to imagine that a six-week class could have much impact, but just last year I got feedback from Rita Charon MD, the director of the Narrative Medicine course, who commissioned a survey of the students’ experience (Miller et al. 2014). It turns out that many of the students see “The City of the Hospital” and the other courses as well as life-changing experiences. To quote one of her respondents: “When you write your admission note …it’s non-fiction writing definitely, it’s just very precise, certain words you must use, certain words you would never use in that note. And then [in] the non-fiction writing [you] put emphasis on different things in a way you could never do in a chart, but they’re telling the same story…. I thought that was directly applicable to what I’ll be doing for the next two years and the rest of my life. Keeping in mind that the admission note doesn’t really tell the full story. It doesn’t tell you necessarily the feeling in the room.”

*

Over the weeks of class, in addition to discussing assigned and student-produced readings, our conversations range onto broader topics. We talk about the ethics of writing about your life as a doctor, not only the concrete issues of how to avoid invoking the wrath of the HIPAA gods but also the ethics of taking notes during your clinical experiences and whether it is exploitative of your physicianly role, or whether it can make you a better physician and should be *required* of medical students. We have lively discussions about whether it is unethical to write about your patients—or perhaps unethical *not* to write about your patients, since all of us as patients benefit from what has been transmitted from all previous doctor-patient interactions, whether in the form of an anecdote on rounds, a medical journal article, an example raised during a classroom lesson, or even a story published in a magazine.

Finally, we touch on a more practical issue, going beyond the writing exercises themselves. How can one be both a doctor *and* a writer? (Hellerstein 2001). In his *Autobiography*, the physician and poet William Carlos Williams (1967) describes his special typewriter that dropped into his desk every time a patient arrived in his consulting room but could pop up again once the patient left. My students compare this to multitasking on their laptops or entering notes into Droids or iPhones—though I do everything possible to prevent them from multitasking during the class itself.

To some degree, I see them realizing by the end of our course, that writing is something they can *do*—a skill like playing jazz guitar or classical violin. But writing is also something they can *be*. It’s a way of experiencing the world, of living in the world of medicine. Living a life of narrative allows one to explore and report upon of the complex worlds of doctoring. Perhaps it helps one to be a humane physician, also a physician-scientist, maybe even a humane physician-administrator, if that is possible. By grappling with the narrative method, they encounter its inherent complexities, the conflicting obligations that come from being both a healer and a bearer of witness. If they are serious about writing, they must honor both their obligations as physicians to preserve confidences as well as their obligations as physicians to reveal truth. There are risks of excessive disclosure and other risks of excessive self-censorship and secrecy. As my students stumble upon these issues in our final meetings, I realize that they have begun the daunting, irrevocable process of becoming permanent residents of the city of the hospital.

* * *

Choosing pieces for this special section was a difficult process, since there have been so many excellent essays and stories written over the years. I reached out to participants from classes over the past several years, based on their final class submissions, which had undergone a process of editing by a fellow classmate followed by rewriting. I also asked them what impact participation in our class had had upon their further medical school careers and whether they had continued creative nonfiction writing. Their brief biographic sketches show how the class has had a continued impact throughout their medical training.

The reader will no doubt be struck by the variety of these pieces, whether in terms of their geographical range, their varied styles and points of view, and their differing levels of literary sophistication. All, though, are heartfelt and full of surprising illuminations, revealing aspects of the medical city that were previously obscure.

- Deirdre Brazil’s “An Initiation” is a moving account of observing her sister’s childhood malignancy.
- Huy Nguyen’s “Blackout” describes observing an eye surgeon working in an isolated Indian slum, and what happens when the power fails.
- Jocelyn Compton’s “Do No Harm” describes her time in a remote Chinese village populated by lepers, and the strange connection she made with inhabitants over cigarettes and wine.
- Benjamin Stix’s “Faces” tells of visiting a childhood friend Matt, who has an autoimmune disease, in hospital, and the heartbreaking experiences of observing how his face changes from one visit to the next.
- Kara Shetler’s “Sundial” takes a different perspective, in telling of an encounter between a depressed patient and two young medical students from the patient’s point of view.
- Tavish Nanda’s “Grey Matter” describes the fascination—and emotional difficulties—of doing research on a database of patients who have died from malignant brain tumors.
- Vivian Ho’s “The Subjects of Science” tells a moving story of the impact of amytrophic lateral sclerosis on a group of small patients she worked closely with in the laboratory.
- Lisa Mack’s “3000 Bolts of Lightning” talks about the odds of a patient getting a rare illness, and her reaction to the strange logic of medicine’s fascination with rarity, topped with a strange personal coincidence.
- Elizabeth Balough’s “King Richard” tells of a rushed, disorienting visit to the Appalachians with a surgical team to obtain organs for transplant from a young man who has committed suicide.

## Essays

### An Initiation - Deirdre Brazil

On every other floor the elevator opened to hallways that were as silent and as sterile as a winter’s morning. But when it stopped on the 6th floor and the doors parted like the red sea, the sounds of shouting, crying, laughing and fighting flooded the vestibule. It was a blast of bustling energy–of mothers fussing over their children like chickens over their chicks, of children fighting over toys like puppies and of people, in general, doing their part to get the show on the road. It strikes the new the same way Ellis Island struck the Irish arriving from plaque stricken Ireland. To the new, the four walls contained an area of waiting and processing but also of foreignness and glittering hope. To the new, a seat in the room was a promise. But to the relapsed, the four walls contained nothing more than the waiting room of MSK’s pediatric day hospital and a seat was just a place to sit.

My sister’s metamorphosis from a high school freshman into a cancer patient was more stunning than a butterfly bursting out of a cocoon because that, at least, is expected. But who expects acute hip pain to turn into a limp that persists for months? And who expects after five visits to the pediatrician and five times returning home with nothing more than reassurance, that he’ll suddenly break down and write a prescription for an x-ray? And finally, who could possibly expect that that x-ray would show a tumor in her pelvic girdle, as clear as the sun rising over a hill?

And that we would become one of the families who sat in the waiting room. I remember scanning it as I sat next to my sister. We laughed when a weak blonde angel decided to alternate between hitting a drum and hitting his Dad’s ankle with a spoon. The father sat back, resting his eyes, and responding to the crisp sound of metal against tarsal with a weakly murmured “ouch.” In response, my mom told us morphine makes children cranky.

What made sense on 68th Street and York no longer made sense when you wheeled your sister off the elevator. My sister thought that the greatest thing in the world was to make someone laugh. With her sparkling blue eyes and radiant face she looked healthy. She didn’t look neoplastic and she certainly didn’t look half as sick as the others. In fact, none of the kids looked like they could, very soon, die. Could anyone die? I didn’t know. Cats and common sense whisper to us that the living can die, but only experience tells you that someone you love can die and you can be forced to live alone.

I remember meeting Michael Reilly in the waiting room. Our moms, with the same drawn faces, figured out that he lived near us in Long Island and that they came from Ireland too. They chatted about back home while he sat deep in his wheelchair and studied the floor. He had a head start on my sister because his cancer fractured his tibia six months before her limp. His mom told us that it happened during a soccer game and he was worried that he wouldn’t be able to play on his team anymore.

Michael told me that people could die, even children. I prayed before him at the wake while his Mom told my Mom about his last night. It was terrible and bloody and they didn’t make it into the hospital until the end. Michael Reilly slept peacefully in his coffin, gone was the discolored skin of toxic treatments and the tired face of constant pain. His younger sister, no more than six, skipped around in her new shoes and twirled to show visitors how her black dress spun out.

Now I am a medical student and I know so much more. I know what small, blue cell carcinomas of childhood look like under a microscope and I know why prednisone treats graft vs. host disease. I know to be suspicious of a fencing injury that progressively worsens after the season ends. And I know that bacteria are racing through the veins of a shivering patient with no blood pressure who tells me she is seeing snakes fall out of the sky. But the more I learn, the more I realize that these answers have nothing to do with my sister. What I need is a textbook that tells me “why?”

* * * *

### Blackout - Huy Nguyen

We stood in darkness. Barely breathing through our masks, we stood unmoving, feeling the sweat crawl over our bodies. By then my eyes had adjusted so I could just make out Abhishek and Sushant, who were standing on either side of me. Abhishek folded his gloved hands together with his fingers pointing towards the floor as if he were nervous, while Sushant held the flashlight steady for Dr. Sinha, the surgeon. The clicks of Dr. Sinha’s tools as he pulled away from the patient were loud, now that both the buzz of the machines and the chatter in the room were gone. I was still shocked that the power went out.

It had been my first time in an operating room. A few hours earlier I had undergone the rituals of scrubbing in and gloving up with ignorant enthusiasm, seeing the excitement and wonder of surgery for the first time with wide eyes. The blackout had yanked me back to Earth; specifically, back to the dusty hot slums of Patna, India. I glanced at our patient and saw her shift her right arm slightly under her gown. Only local anesthetic had been administered, so even though she couldn’t feel pain, she was still very much awake. She must have realized we had stopped and heard our silence. I wondered if she knew there had been a blackout, and whether she was panicking after recognizing her surgery had been halted. I couldn’t see inside her mind, though, no more than she could see us.

It had only been about one minute since the blackout, but I did not know how much longer I could last. Still, no one had said a word. My shirt clung harder to my back beneath my gown, but I could not air it out since my hands were trapped beneath sterile gloves. I shifted my weight to my right foot with my left knee bent slightly, and breaths came hot and heavy under my facemask as I struggled with the weight of the air. I looked back at Dr. Sinha, who had just finished the careful extraction of his tools from our patient’s eyes under Sushant’s flashlight. He didn’t reach for any other tools. Our patient had stopped moving her arm, but her slow and steady breaths reminded us that she was still there.

There were two kinds of cataract surgeries which I had seen Dr. Sinha perform that day. The older method, ECCE, took longer and required a traditional incision in the eye. The newer one, Phaco, took only a few minutes but required an advanced machine. That day, Dr. Sinha started with a few ECCE cases to show me what he used to do before his clinic obtained a Phaco machine, but since noon he had switched to only doing Phaco. As we stood there in the dark, I looked back at the tools on the green surgery tarp. I knew Dr. Sinha could finish the case with an ECCE incision, but he remained motionless, slumped forward with his elbows on the edge of the operating table. Once we’re spoiled by technology, it’s hard to go back, I guess. I felt a bead of sweat snake down the small of my back. Our patient shifted her arm again. She must be feeling the heat too, but I hoped she could put up with it until the lights came back on. She had trusted her doctor to restore her sight, and her doctor had trusted the machines to work for him. But now the machines were down, and he had chosen to wait. The rest of us were now nothing more than silent ghosts in the darkness, hovering, staring. I looked to Abhishek, who still had his fingers pointing toward the floor. He closed his eyes.

* * * *

### Do No Harm - Jocelyn Compton

He props a splintered hoe against the ravine and offers me a cigarette. These gestures have become our common language.

“Hun hao,” I say. He smiles and returns, “Hun hao, hun hao.” Very good is one of the two phrases I know in Mandarin. Of course, he doesn’t speak Mandarin either, but a distinct dialect. Bu hao is the other phrase we share. Not good.

The tattered box of cigarettes most likely came from the market thirty miles away. The outdoor market has one booth packed with tobacco, opium, and other herbs for good health. With this cigarette, he is wishing me a long life, many children, relaxation, freedom from illness.

It’s only March, but the mid-morning sun is blistering. Villagers and college students survey the dry cracked countryside together, waiting for another delivery of dusty gravel to arrive. Some lean against shovels, hoes, and other agricultural tools. It took three days of work just to flatten a winding path into Xiao Shui Tang; it would take another seven to spread the makeshift pavement over it.

The villager’s only hand is occupied with returning the package of cigarettes to a buttoned pocket, so I strike the match for us. Here’s to good habits and great health, I think and take a drag. Looking around at the workers, it seems that there are about sixty members of the village, but only forty whole human bodies. Fingers, toes, arms were each slowly lost to leprosy over the years. Dignity, purpose, self-worth seemed to vanish with them. In the ‘sixties, the government prescribed exile for those afflicted with Mycobacterium leprae, the bacteria that causes Hansen’s disease. And here we are, in Xiao Shui Tang, in 2008.

At night we slept on a flattened hilltop in the middle of the village, which served as the hospital compound. The doctor’s hut, a one-room establishment on the compound, was stocked not with antibiotics and multidrug therapy that the villagers desperately needed, but rather rice wine. As the sun set and villagers came in from the fields, many crawled to the doctor’s home seeking rice wine to help soothe their sore appendages.

“It doesn’t cure them,” said Dr. H, the village doctor. “But it takes off the edge. What else can we do? Cigarettes are too expensive to give to everyone.”

Some years before our cohort arrived at Xaio Shui Tang, another American volunteer group had delivered prosthetics to the village. Villagers who had been immobilized returned to the fields and rejoined their small agricultural society. The plastic appendages had saved many of them from suicide.

But now, the ill-fitting prosthetics often eroded limbs and incited angry infections. Red, poisonous, hungry lines raced up numb, swollen legs. The prosthesis that had breathed life back into a dilapidated body was now suffocating it.

One evening I sat with Dr. H. His first patient of the evening was about sixty years old. He had been living in Xiao Shui Tang for most of his life, and had barely caught a taste of the outside world before Mycobacterium leprae had sentenced him to a life on the outskirts of society.

When the man removed his worn plastic leg, a putrid smell filled the small hut. I looked at the festering stump.

I was afraid.

Over half the village had died since the last volunteer visit. Blisters became deadly infections, claiming shaking, feverish, exhausted villagers. No amount of rice wine or cigarettes could cure them.

Half a world away, carelessly forgotten in a college dorm dresser, I thought about an unfinished course of ampicillin: a likely antidote for the villager’s infections. A simple solution, out of reach.

But how can you know if you’re truly doing good? What if the cure leads to a more vicious problem? How do we understand the consequences of our intentions and actions? And where do you begin, if the only way to express compassion and concern is bu hao?

Out on the road, we wait together in silence. When will the gravel arrive? Our precious cigarettes are just about spent.

* * * *

### Faces - Benjamin Stix

My heart always starts to beat faster as I press the button to the 7th floor in the Heart Hospital. Today, Matt texted me in his usual way, “Whatsup dude”–a sort of open ended, no-pressure request that I come visit him in his prison on the north side of the unit. As I get off the elevator and walk slowly down the hallway, I always glance at who is at the nurses’ station, worried that I will see a professor who has lectured me or a fellow med student, a bit older, reading over charts or bent near an attending, learning how to write up a patient or assess a case properly. I stroll past, outwardly nonchalant, but inwardly in tumult as I hang a right towards Room 150.

Each time that I visit Matt, I first look at how his face has changed since I last knocked on his closed hospital room door. He has been in the hospital since January 15th–trapped for the past 4 ½ months. Matt’s heart failed abruptly, his antibodies attacked him furiously, and he landed up in the CCU, with an LVAD and an RVAD pumping his blood for him. Now Matt awaits yet another transplant.

I always look at Matt’s face, not just because everyone looks at faces first, but because Matt’s face ebbs and flows in a bizarre way. In high school, Matt was diagnosed with a particularly pernicious form of scleroderma that attacked his organs and obliterated his heart. Somehow, he managed to graduate and went on to a degree in engineering, and by sheer force of will he survived until his first transplant. However, the scleroderma had left its mark. His face was drawn and skeletal because of the lack of collagen, and a large scar from his transplant ran down his chest. But the new heart drastically improved Matt’s life, and immunosuppression seemed to keep his scleroderma at bay. He enjoyed online dating, obsessive nutrition, weight lifting, and what he described as “the art of picking up women.”

But five years later, Matt was suddenly in hell again. His overloaded immune system may have contributed to his abrupt turn, but his heart was in rejection for unknown reasons. When he first got his VADs installed, Matt looked utterly haggard, worse than I had ever seen him; he was 110 lb and 6′ 1″, on fluid restriction to prevent the edema that was one of many symptoms.

Today he looks better than the last time I came to visit, some color and form have returned to his skeleton, and his head sort of resembles a pear. He has unwillingly shaved his scalp due to a chemo medication he was on, and his hair is coming back in light flecks, gradually getting thicker. But his cheeks still have an odd droop to them, the extra fluid in his face is dragged down by gravity into a dead-end, unable to leave and go to his bladder.

I always have this profound sense of guilt whenever I visit Matt, because I can leave.

My life isn’t in stasis, while the only thing that changes in Matt’s room is his face. I try to talk about what I am learning about in school, my girlfriend and our long distance relationship, my feelings about the news, but worry that I sound stilted. I can have this comparatively easy life, while a friend whom I have known since I was five years old is dying.

Once or twice a week, I join him in counting out the minutes until some kid gets drunk, crashes his car, gets brain dead, and donates his heart.

In immunology, I learn about transplants on a macro level. I can tell you the average rates of transplant rejection of various organs at one, five, and ten years for hearts. But Matt is not average, is he? What if he’s worse? Or could he be better? As a first-year medical student, I know more than I should about his condition, but not nearly enough to help my friend. It is worse than sheer ignorance.

This visit, Matt tells me about his collaboration on a book with a woman he hired from Craigslist. The book starts out as Matt’s biography since his initial diagnosis, but his goal is to switch gears mid-book and write a field guide for someone diagnosed with a chronic illness, a step-by-step manual for someone who is also stuck in the hospital for a long period of time. Matt has always been painfully smart, but we share a certain degree of laziness; outsourcing his work and his story seems appropriate. But it also occurs to me that he doesn’t really have the energy to undertake a task like writing a 200-page memoir. I also think his profound sense of denial and minimization of his illness also might paralyze him to really write much about himself. Better to let someone else capture his life, and he can reap the benefits.

So I don’t say much beyond platitudes about his book project, such as “Sounds great!” or “What an original idea!” And I get up uncomfortably to go, never knowing whether I should shake Matt’s hand or not, worrying that I might infect him with something that sends him over the edge, changing his face into a death-mask.

* * * *

### Sundial - Kara Shetler

“The students are here for you,” said the nurse as she opened the door to my room, flattening her back and outspread arms against it to let me pass. I noted with my usual admiration how her bearing left no room for uncertainty. Rousing myself from beneath the weight of ticking seconds, I floated past, all eyes and brain.

By the nurses’ station stood two young women in short, white, wrinkled coats. They wore their eagerness like perfume; I wondered vaguely if the haze gave everything they saw a certain tint, as the dust in a sunset sky. The nurse unlocked the double doors decisively and we went through, down the halls and out the doors of the Psychiatric Institute, across the curved street under the mid-afternoon shadow of a building and finally through its doors. My guides seemed borne along on a kind of swirling, buoyant fizz. I felt foolish; I tried to decide if the feeling was for me or for them.

We entered a tired classroom on an upper floor. A circle of similar white coats beneath similarly fresh faces sat around the periphery in flimsy classroom seats attached to inadequate desks. A black and white institutional-grade clock reigned over the room from high on one wall; light diffused in through the large windows on another. The room looked out on an apartment building, eye to eye with its tawny crown of molding that marched across a vividly blue sky in relief, bathed in the sun from behind us.

“I’m Dr. D.,” said a man who was not wearing a white coat. His shirt-sleeves were rolled up, and he extended an easy forearm to shake my hand. “I’m the resident working with these medical students. Thanks for coming to talk to us today.” He looked almost as young as the rest, but I noticed flecks of gray in his hair. His face wore something between openness and businesslike detachment. It was a face I knew well.

I sat in a chair placed in the front of the room, this one without a desk, and settled into the stream of seconds that flowed from the clock-face above. I felt the balance of foolishness tip in my direction. The perimeter of faces turned toward me were blank, their only task to listen. I wondered if this was degradation but couldn’t really care one way or another. I cast my mind around for a story of who I was in this room, some kind of anchor or point of reference. The young doctor with the rolled sleeves seemed the most solid bet.

“So Mr. T,” said one of my student-interviewers, her eyebrows and the pitch of her voice both elevated. “Can you describe what you mean when you say your depression is severe?”

I gazed out the window at the crowning stone, bold in the sun and sky. It looked like a picture meant for a wall or a book. It hit me, bright and heavy, in the chest. I turned back to the faded room and the flow of the clock.

“I just can’t seem to fill the day,” I answered.

* * * *

### Grey Matter - Tavish Nanda

“Every single one of them dies. They’re all dead,” Dr. T paused for a moment, “Yes, they’re all dead,” he confirms. He scrolled down an excel spreadsheet, the top of each column color-coded, arranged side by side like crayons. Infinitely long.

On the one end were patient names. Stacked alphabetically, like a grocery list.

We never used them. Each person little more than a nine digit code.

*

My research partner and I sit next to each other, surrounded by four computer screens, alone in a cold, quiet room. A ghost department past five p.m.

“Next?” he calls.

“143-90-6621.”

“Location?”

“Anterior Falx,”

“Where’s that?” he asks.

“I have no fucking idea.”

We pore over an MRI, sifting through layers of brain matter, memories, experiences, love, hate, sin, thoughts, regrets, passions, reduced to grey mush behind pixelated glass.

“Piece of shit image,” he says.

“Yeah.”

Our eyes dart back and forth in unison. Peering wildly.

“Come on, guy,” I say, talking to the patient that once was.

“There, he’s got one right there,” I jump. My index finger prods the screen.

“No go back, just…right…you see it? That little smudge.” I gleam.

He leans in, squinting his eyes. A light grey orb pops out from between layers of normal matter. The eight ball of death.

“Yeah that’s it, got to be, what do you think?” He moves the mouse back and forth.

“I’m 100 % positive,” I say.

We take the measurements.

“Coronal?”

“14.3”

“AP?”

“7.2”

“Trans?”

“4.44”

“4.44?”

“Yeah.”

The spreadsheet did the rest. Spitting out a tumor volume.

“Did it fail?”

“Don’t they all?” I joke.

We laugh for a moment. He shakes his head with a delayed sense of ethics.

“That’s so bad…” he forces, as if stopping his fall from grace, “did the treatment control it?”

“No, it grew…does he have a follow up?” I ask.

We pillage through more medical records and find a second MRI, three months post-procedure.

It loads slowly, real slowly.

“How was your weekend?” I ask.

“Good, went to this bar in Brooklyn called Union Pool.”

“Hipsters?”

“More like rich white kids pretending to be poor,” he says.

“So, hipsters,” I conclude.

The image finally loads. He clicks the radiology report first.

“They had a special…one beer and a Jager shot for three dollars,” he says.

“Stop right there. What does it say…?” I interject.

*Eight new enhancing lesions were recognized compared to previous imaging (4/3/12) possible recurrence or expansion of the…*

“Did you say three dollars? That’s fucking amazing.”

He thumbs over the image. Shifting through the slices. The eyes that saw the love of his life, the death of his father, the beauty of a New York sunset, the face of his oncologist, only a little while ago. The nose that had smelt Chinese food, or perfume, or the air after a heavy rain.

Within the masses of his mind we find a new peppering of blotches, ink stains almost, half of dozen, their edges rugged…alien even.

“Holy shit, it exploded,” I say.

“Six more.”

“Goddamnit, we’re going to be here forever if every one of these people have multiple tumors.”

“I want to make meal plan,” he says, irritated, peering down at the time. It’s 6:30 on a Monday, and dinner is more concerning than death.

I add six more columns under the name. Finally, I look at it.

*Salvarado.*

“I think this guy was Hispanic,” I mutter.

“Hm?” He asks.

“Nothing,” I say.

“Who’s next?”

I scroll to the next line.

“557-44-3326”

*

One month ago, a hundred records sat on a conference table. Each had a picture. A smiling face of some patient clipped against the top of a vanilla folder.

“Jesus, are all these people dead?” I ask.

“Unfortunately,” Dr. A says.

“Why do they have to show us the pictures?” I ask.

“They change a lot during treatment, it’s to help, keep everything straight.”

“That’s a little morbid,” I say.

My research partner storms into the room.

“Sorry I’m late,” he hangs his head low, shamefaced, before taking a seat.

“I want to bust out three papers by the summer, so let’s try to get this done in the next few weeks, and burn through some abstracts. These are the only paper records, the rest are in the database, did you guys get access?”

We both nod together.

“I think we have about three hundred patients, that’ll be at least five hundred tumors. That’s more than any other institution!” he claps his hands triumphantly, “No other institution has that kind of volume.”

Dr. A is always energetic, moving from side to side, like a Ritalin-infused child. We run through the project one more time. The nuances of brain metastases, what they look like, the prognosis. He tells a story about a patient who’s lived eight years post-procedure.

“Eight!” He exclaims, “and is still alive!”

Midway through he gets paged and blitzes from the room like flash, the superhero. He’ll reappear, an apparition overseeing our progress, from week to week, giving a heartfelt high five.

“Aight boys gotta go, a young Korean lady with a MASSIVE met. Whole temporal lobe.”

“Good luck,” we’d both say, not really knowing how to respond.

We split the files in half. I move from one to the next. So many histories, so many faces. I can’t help but imagine seeing each of these people in a grocery store. Maybe I did one time. Maybe this is the circle of life, and a certain picture was meant to fall into the hands of a much-too-young medical student. But I recognize none of them. And with time I forget they’re even people.

I turn to my colleague.

“Can you imagine all these people were alive?”

“Well yeah,” he says, unfazed.

“No I mean, you know, like alive. They all had families, or fought in a war, or something, you know?”

“You don’t know that.”

“I know I don’t know that. I’m just saying…never mind. It’s sad they all died.” I say, with more amazement than sympathy.

“Yeah,” He replies.

*

An MRI of the brain is like seeing the Earth from the international space station. You can observe the capsule as a whole, imagining all that is contained within. All the tiny, fleeting moments, the daily daydreams, the lives of a thousand fictitious characters. A home to seven billion decisions. You don’t see any of them, but you know they existed at some point. Existed to be more than data points on an Excel spreadsheet.

* * * *

### The Subjects of Science - Vivian Ho

I steadied the tray, inhaled, and opened the door. The cell reeked of urine and astringent. He slept fitfully under the tepid fluorescents, his bloated belly heaving over withered, useless legs. When I pressed on his abdomen, pockets of air and undigested food wriggled underneath.

No one else in our study had degenerated so quickly. Amyotrophic lateral sclerosis, colloquially known as Lou Gehrig’s disease, causes ascending paralysis at variable rates. In five weeks, we had lost his legs and intestines–his lungs, the fatal choke point, would be next. His plight echoed the devastation of the disease, and the hope my supervisors had for our experimental treatment.

I loaded a syringe with one hand, feeling for a soft spot with the other. Months of daily injections had stippled his skin with tough, fibrous scars. At one point, he woke with a shiver, regarding me with cloudy red eyes. In one fluid, practiced movement, I lifted the mouse, tucked his tail aside, and injected a control solution into his upper hip.

Human illness engages us physically and philosophically. In addition to our symptoms, we feel a sense of cosmic misfortune. We wonder why: “Why me?” or “Why not someone else?” In the realm of animal testing, the answer to these questions is clear. Mice, rats, and rabbits are sick because we design them to be. We instigate mutations and breed for the trait. As an intern in a mouse laboratory, I learned how simple answers gave way to murkier ethical concerns.

Lou Gehrig’s disease in mice is both identical and foreign to the real thing. In both parties, the paralysis starts at the lower limbs and inexorably rises. As the mice weaken, their fur becomes matted, their paws shrivel, and their food has to be moistened to be digestible. However, mice and humans with Lou Gehrig’s disease experience very different treatment. People receive ventilators and wheelchairs. Mice get a daily dose of experimental drug, a grueling regimen of physical testing, and 120-day expiration date. After 120 days, it is considered cruel to keep them alive.

I administered injections and put the mice on treadmills, balance beams, and in swimming pools to quantify their decline. It was dull, time-consuming work, requiring hours of prodding paralyzed mice over narrow ledges and fishing them out of swimming pools. What kept me going was not a desire to publish, but a nagging sense of humility and shame. No matter how terrible my day could be, I was always the least miserable animal in the testing room.

My views on animal testing are ambivalent. I feel strongly about its contributions and the need for ethical restrictions, but these opinions feel divorced from the reality of everyday science. Researchers aren’t actively trying to be cruel to animals, but we do feel compelled to suppress our empathy at times. At best, animal testing feels morally permissible, but not comfortingly right.

When the mice reached their 120th day of life, another intern filled their veins with formaldehyde and removed their brains. The brains were frozen, then sliced with a $400,000 machine into 2-micron-thin salami slices. The salami was stained with antibodies, washed, and then mounted on slides. Other research assistants would count the stained cells, and a report filled with numbers would appear on my principal investigator’s desk.

At the end of my internship I thumbed through our report, resplendent with graphs and slides. It was impressive, particularly because I felt like I had nothing to do with it. Somewhere, the smells, splashes, and squeaks of science had been covered up by *p*-values and literature reviews. My name was in the acknowledgements, but there was little respect paid to the hundreds of little lives lost. If you look closely, you’ll find them in parentheses, after the “n=” sign. But whether they belong there is up to you.

* * * *

### 3000 Bolts of Lightning - Lisa Mack

The chances of getting cholangiocarcinoma: 0.002 %.

The chances of developing pancreatitis after a diagnostic endoscopic retrograde cholangiopancreatography: 4 %.

The chances of gastroparesis after a pancreaticoduodenectomy: 25 %.

This man was one in a million. Technically speaking, he was one in 2.5 billion, depending on your sources, of course, but needless to say he was not lucky. That’s not even taking into account the fact that he was about to have a bumbling first-year medical student poke and prod the few parts of his body that remained healthy.

He was a middle-aged math professor, and (as we learned obtaining his travel history) loved visiting Italy. Medically speaking, he had done everything right. He worked out consistently, adhered to a low salt, low fat diet, eating lots of vegetables, had never smoked, and his drug of choice was a weekly glass of wine with his loving wife. Even his family was perfectly healthy, no illnesses: not a sibling with high blood pressure or an aunt with diabetes. And definitely no cancer. His dad lived well into his nineties, and his mom was still going strong, nearly 100. But I guess it doesn’t matter when you’re unlucky.

The chances of getting struck by lightning are about 1/750,000, over 3000 times more likely than this man’s past month of misfortune. I’ve always thought it was funny that people compare everything to the chances of getting struck by lightning as opposed to anything else, but it’s one of the few statistics I know without having to look it up. My great-aunt, a near urban legend in my family, like this man, was also unlucky, maybe 3000 times luckier than him, but I guess in the end that didn’t give her any advantage. Lightning strikes when it wants to.

I felt bad bothering him. I felt even worse when I quickly learned that he and his wife were delightful. She mostly kept to herself, occasionally chiming in with a helpful detail, but she was absorbed in her phone. I imagine she was sending updates to family and friends, probably littered with phrases like “We’re hanging in there!” and “Things are going well!” even though her tired face showed she knew that wasn’t true. He was quiet, uncomfortable, but still able to muster a genuine smile (albeit a weak one) when he talked about his children.

I progressed through the physical exam, pulling shiny instrument after shiny instrument out of my Mary Poppins carpet bag of a white coat. When I was done I threw the tools into my backpack. From head to toe I converted this man’s story into a series of values: 3+, 123/85, 86, 5/5, etc.… and with each one my pockets got a little lighter.

A friend later told me I was lucky to get a patient with such a unique case, someone so interesting. Her patient “just” had a heart attack. To be honest I didn’t know anything about the rarity of this cancer versus that one. Hell, I could barely pronounce what he had. But I remember thinking: isn’t every patient one in a million? One in seven billion, or whatever the growing number is now? Sure, some diagnoses are made more than others, but every trip to the hospital is a one of a kind story. The fact my patient had a rare cancer wasn’t ‘interesting’ or ‘cool’ to me. It was sad. Not necessarily more sad or less sad than a heart attack, but sad. It’s all just sad. The fact that this math professor got his life-changing diagnosis on March 14th, Pi Day, however? That was interesting.

The exam was over, the preceptor was gone, and the final glob of Purell was evaporating off my hands. I was no longer a ‘future doctor’, just a girl who happened to be wearing an oversized coat with too many pockets, in the room of an unlucky man.

“Where in Italy did you go?”

“A small town in the north, an hour or two outside of Venice, most people have never heard of it.”

Turns out I had heard of it. Turns out not only had I heard of this small town of only a few hundred, I had been there myself. At the exact same time as him and his wife. Now what are the chances of that?

* * * *

### King Richard - Elizabeth Maier Balough

Settling down on the plastic sofa bed in the office, I waited for the sound. The hospital felt so urban at night, the lights all-aglitter and everyone bustling round. I couldn’t rest, not only because of my anxiety over what was about to ensue, but also because this is simply not a resting place.

The pager went off, and my hands started to shake. At least when I called back the number, the coordinator was gentle with the information. She wanted to let me know in advance that it had been a suicide. I thought I heard her smile on the other end of the line. Estimated time of departure would be 4:00 a.m. Did I want pancakes on the plane, or something else? I’ll pass this time, I said.

Where were we going? I couldn’t remember. As usual, I struggled with the lock on the supply closet door. Practically sweating expletives I finally felt the key twist, and I tumbled into the small room propelled by the force of my frustration. My compulsive tendencies revved up as I packed the big yellow bag and cooler for the trip. I checked the contents twice, no, three times, against the list and then put one extra of everything we could possibly need on top. Into my back pocket I stashed three kits for drawing arterial blood gases because the junior surgeon was sure to drop one during the procedure, and pulling out a spare and handing it over, sterilely, at just the right moment was the one thing I could do during the whole trip that would make me feel like I had any business being there.

At the distant hospital nestled somewhere in the Appalachian hills I met him. He wore a paper crown. Written on it in a shaky hand was this: “King Richard, who held the weight of the world upon his shoulders.” His eyes were covered with the gauze that covered the hole he had made in his head with his dad’s shotgun. From the nose down, though, his body was intact, almost vibrant, like Christ in Rembrandt’s *The Storm on the Sea of Galilee*. The phrase the doctors used was “well-perfused.”

We took his organs and then we were out like bandits, running down the dim halls of the country hospital, jumping into our getaway car, the ambulance with its screaming sirens, and then into the little plane and up, up into the air amongst the angry clouds and the sunset. All in the service of someone who needed a new heart.

Or was it for that person? Maybe it was for us. Look at what we can do.

And as for King Richard? Well, medicine hasn’t advanced far enough to save the likes of him, I guess.

## Author biographies

### Elizabeth Balough

Elizabeth is currently a second-year student in the MD-PhD Program at Columbia College of Physicians and Surgeons. She became interested in the intersection of writing and medicine as an undergraduate at Columbia, where she was introduced to the essays of Walker Percy. The writing course that was part of her first year as a medical student reinvigorated Elizabeth’s interest in using creative non-fiction as a means for critically processing her experiences in medicine and science. Elizabeth studies the neural circuits underlying emotional learning and memory, and she hopes to become a practicing psychiatrist, researcher, and writer someday.

* * * *

### Deirdre Brazil

I am a family medicine resident at Hunterdon Medical Center in Flemington, New Jersey, and I am also training as a physician acupuncturist. I am finishing my chief year in June, and I used some of my academic time to create a narrative medicine lecture series that was based on Dr. Hellerstein’s “The City of the Hospital” class. It was very well received, and I plan to continue to develop the lecture series during my final year of residency. I have continued to write, and I consider narrative medicine to be a great source of emotional strength for me and an integral part of my development as a physician.

* * * *

### Jocelyn Compton

I’m a graduating medical student at Columbia University’s College of Physicians and Surgeons. This past March, I matched into orthopaedic surgery! Before medical school, I attended Yale University where I participated in a number of outreach opportunities in China, Nepal, and various other foreign countries. These service trips affected me deeply, and engrained in me the importance of meaningful, appropriate, durable intervention. I believe that only through thoughtful reflection and writing did I come to fully understand the implications of our best intentions. For me, revisiting words I have written detailing my experiences has been immensely helpful in achieving insight into my beliefs about the purpose of medical care and what it means to “do no harm.”

* * * *

### Vivian Ho

I am a second-year medical student at the Columbia University College of Physicians & Surgeons. I received my undergraduate degree in Biology at Stanford University, where I also enjoyed taking writing classes and teaching science in low-income schools. While I have yet to narrow down a specialty, I gravitate toward teaching and academic medicine.

Dr. Hellerstein’s “The City of the Hospital” course was foundational in building regular writing habits. Ever since I started on the wards, writing has become an invaluable method of organizing and processing my reactions. Even on my busiest days, I strive to take a few minutes to jot down my thoughts. Finding my voice on paper has been the difference between surviving and thriving in medical school, and will no doubt continue to be a source of insight, perspective, and comfort throughout my medical career.

* * * *

### Lisa Mack

I’ve always been surrounded by the sciences, having majored in chemical engineering and worked as a software engineer before coming to medical school. I never would have thought that as a medical student I’d be writing (or be doing much of anything outside of studying for that matter), but I realized only a few moments into this new experience that I had landed at an institution that emphasized the importance of various methods of self-expression. I joined the medical school theater troupe and performed with other groups: all amazing experiences, but all with the comfort of borrowing other’s words, so when I was placed into the non-fiction writing narrative medicine class I was terrified. I was surprised to learn, however, that I loved writing. It wasn’t a distraction from medicine like I expected it to be, but rather changed the way I interacted with my patients. Writing about my exchanges served as a reminder that patients are so much more than their illnesses, and to be successful at my job as a student I have to understand the whole story, not just the lab values. Even though the class ended almost a year ago I’ve kept writing, whether a story or a few scribbles, just to make sure I don’t forget this.

* * * *

### Tavish Nanda

Originally from the San Francisco Bay Area I attended the University of Southern California, majoring in anthropology with a minor in screenwriting. In college I interned at NBC, the *Huffington Post*, and the *Japan Times* before applying and eventually attending medical school. I have an interest in narrative medicine and film with the eventual goal of combining those passions with a career in medicine. “The City of the Hospital” course helped provide a structured environment to mold a writing style and explore creative potential within the medical field. The course, being taught as part of a medical education curriculum, helped define the often-amorphous idea of the “physician writer,” making it seem like a more attainable reality. Since taking the course, I’ve continued writing short pieces of a similar fashion, with the hope to continue doing so into residency, and as a professional.

* * * *

### Huy Nguyen

I grew up in California and studied Chemical and Physical Biology at Harvard before coming to Columbia for medical school. As a son of immigrant parents, I was always aware of cultural differences and the general world around me. My interest in writing down my observations began as a child when I received a small journal as a gift. Over time, my journaling grew from recording daily activities and travels to writing my impressions and thoughts about academic and social issues. When I took Dr. Hellerstein’s “The City of the Hospital” class as part of the Narrative Medicine course in medical school, I realized that journaling my impressions on the wards could reveal what was most important to me in medicine, and consequently influence what qualities I would have as a physician. As I went through my medical training, I journaled stories about my patient interactions, which made me reflect on not only how they made me feel but also how I thought the patients felt. As I move forward, I believe that by writing my own stories, I could learn better how to listen to those of my future patients.

My interest in ophthalmology began in undergrad when I traveled to India to volunteer in an eye clinic. I solidified that interest during a research year in medical school where I investigated gene therapy on patient-specific stem cell-derived retinal cells. I now have matched into Harvard’s ophthalmology residency at the Massachusetts Eye and Ear Infirmary for the next step of my training as I pursue my goal of becoming a retinal surgeon. I enjoy playing tennis, photography, and exchanging good stories over a home cooked meal.

* * * *

### Kara Shetler

Kara was a writer long before she decided to be a doctor, publishing her first short story in the local newspaper in third grade. She majored in English as an undergraduate while completing her premedical coursework. As a medical student, she served as the editor-in-chief of the 2013 edition of *Reflexions*, the art and literary journal of Columbia University Medical Center, and participated in a pilot project introducing a longitudinal writing component to the medical curriculum. She was inspired to continue writing by “The City of the Hospital” class and has found this practice to be one of the most useful tools for processing the challenges and joys of medical training. She plans to pursue a residency in either internal medicine or neurology.

* * * *

### Benjamin Stix

Benjamin Stix grew up in Washington Heights, New York. He comes from a family of journalists, so his apartment growing up was stuffed with magazines and newspapers. He initially became interested in writing in high school and college, but took a detour into the world of business, before deciding on medicine as a career. The narrative medicine class “The City of the Hospital” was the first time he had explored deeply personal non-fiction writing. He hopes to continue writing in this style throughout his career. He is currently interested in the nexus between intensive care and palliative care, and he has undertaken a research project in the Department of Anesthesiology on reasons for consultation of palliative care in the ICU. Benjamin and his wife, Annesly, are expecting their first child in July, 2015.
